# Relationship Between Epicardial Adipose Tissue and Atrial Fibrillation in Heart Failure With Preserved Ejection Fraction

**DOI:** 10.7759/cureus.80827

**Published:** 2025-03-19

**Authors:** Oguzhan Yucel

**Affiliations:** 1 Cardiology, Buyuk Anadolu Hospital, Samsun, TUR

**Keywords:** atrial fibrillation, cardiac remodeling, echocardiography, epicardial adipose tissue, heart failure with preserved ejection fraction

## Abstract

Introduction and aim: Heart failure with preserved ejection fraction (HFpEF) is a significant clinical challenge, often coexisting with atrial fibrillation (AF), which exacerbates patient outcomes by increasing risks of stroke, hospitalizations, and mortality. Recent studies suggest that epicardial adipose tissue (EAT), a metabolically active fat depot, may contribute to AF pathogenesis by promoting atrial remodeling and fibrosis. This study aimed to evaluate the relationship between EAT thickness and AF in HFpEF patients.

Materials and methods: A total of 110 HFpEF patients were included, with 20 (18.2%) having documented AF. EAT thickness was measured using transthoracic echocardiography, and AF was confirmed via electrocardiography.

Results: Patients with AF had significantly greater EAT thickness compared to those without AF (8.3 ± 0.9 mm vs. 7.1 ± 0.8 mm, p < 0.001). Receiver operating characteristic (ROC) analysis demonstrated that EAT thickness was a strong predictor of AF (AUC = 0.87, p < 0.001), with a cut-off value of 7.5 mm achieving 89% sensitivity and 75% specificity.

Conclusion: These findings indicate that increased EAT thickness is independently associated with AF in HFpEF patients, highlighting its potential as a biomarker for AF risk stratification. Future studies should explore whether targeting EAT could improve clinical outcomes in this high-risk population.

## Introduction

Heart failure with preserved ejection fraction (HFpEF) is a common and complex clinical syndrome, accounting for approximately 50% of all heart failure cases. It is characterized by normal left ventricular systolic function but impaired diastolic function, leading to elevated filling pressures and symptoms such as dyspnea and fatigue [1,2]. HFpEF is associated with substantial morbidity, mortality, and frequent hospitalizations, with its prevalence rising due to aging populations and increasing rates of comorbidities such as obesity, diabetes, and hypertension [3].

Atrial fibrillation (AF) is a frequent comorbidity in HFpEF, occurring in up to 40% of patients, and is associated with worse clinical outcomes, including higher risks of stroke, heart failure exacerbations, and mortality [4,5]. Beyond these adverse outcomes, AF is also a significant predictor of coronary artery disease (CAD) and myocardial infarction (MI), as accumulating evidence suggests that AF and CAD share common pathophysiological pathways, including endothelial dysfunction, systemic inflammation, and atrial remodeling [6-10]. Additionally, AF is increasingly recognized as a major risk factor for cognitive impairment and dementia, likely due to cerebral hypoperfusion, microembolization, and systemic inflammation [11]. The intricate interplay between HFpEF and AF is multifactorial; hemodynamic alterations such as left atrial pressure overload and atrial fibrosis in HFpEF predispose patients to AF, while AF exacerbates diastolic dysfunction and left ventricular filling pressures, creating a detrimental cycle [12].

Emerging evidence suggests that epicardial adipose tissue (EAT) may play a pivotal role in atrial remodeling and AF pathogenesis. EAT is a metabolically active fat depot located between the myocardium and visceral pericardium, with both direct anatomic and paracrine effects on the myocardium. It secretes pro-inflammatory cytokines and profibrotic mediators such as interleukin-6 and tumor necrosis factor-α, which contribute to atrial fibrosis, conduction abnormalities, and arrhythmogenesis [13,14]. Increased EAT thickness has been associated with both the development and severity of AF, making it a potential biomarker for risk stratification [15].

In addition to EAT, systemic inflammation is increasingly recognized as a key contributor to AF pathophysiology. High-sensitivity C-reactive protein (hs-CRP), a marker of systemic inflammation, has been linked to both AF incidence and adverse outcomes in AF patients, including thromboembolic events and cardiovascular complications [16,17]. Given the pro-inflammatory state in HFpEF, particularly in the presence of AF, hs-CRP may serve as a critical biomarker for disease progression and therapeutic targeting.

Although the relationship between EAT and AF has been well-documented in the general population, limited data exist regarding its role in HFpEF patients specifically. Considering the unique pathophysiological features of HFpEF, including systemic inflammation, atrial remodeling, and heightened cardiovascular risk, understanding the contribution of EAT in this population is of particular importance. This study aims to investigate the association between EAT thickness and the presence of AF in HFpEF patients. By identifying EAT as a potential biomarker and therapeutic target, this research seeks to provide new insights into the underlying mechanisms of AF in HFpEF and inform strategies for risk stratification and intervention.

## Materials and methods

This cross-sectional study was conducted at Cumhuriyet University Faculty of Medicine Hospital, Turkey, to investigate the relationship between EAT thickness and the presence of AF in patients diagnosed with HFpEF. A total of 110 patients were included, all of whom met the diagnostic criteria for HFpEF based on current guideline recommendations. The inclusion criteria required patients to have a left ventricular ejection fraction (LVEF) of 50% or higher and clinical symptoms of heart failure such as dyspnea, fatigue, exercise intolerance, and echocardiographic evidence of diastolic dysfunction. Diastolic dysfunction was defined by parameters such as an elevated E/e' ratio greater than 14, left atrial enlargement, or increased left ventricular wall thickness. The diagnosis of HFpEF was confirmed by a cardiologist experienced in heart failure management. Patients were excluded if they had a reduced LVEF (less than 50%), significant valvular heart disease, recent MI, known inflammatory or autoimmune conditions, active malignancy, or a history of cardiac surgery within the past six months. In addition, patients with poor echocardiographic image quality that precluded accurate measurement of EAT were excluded from the analysis. Patients with uncontrolled hypertension or thyroid dysfunction were also excluded to avoid confounding factors that could influence the results.

Baseline demographic and clinical data were collected for all participants through detailed medical record reviews and patient interviews. Variables included age, sex, and the presence of comorbidities such as hypertension and diabetes mellitus. Blood pressure measurements were taken using a validated sphygmomanometer after five minutes of rest in the seated position. The presence of AF was confirmed by a standard 12-lead electrocardiogram (ECG) performed at rest or by a documented history of persistent or paroxysmal AF in the medical record. Persistent AF was defined as AF that required medical or electrical cardioversion, while paroxysmal AF was defined as AF episodes that terminated spontaneously within seven days.

EAT thickness was measured non-invasively using transthoracic echocardiography. Experienced echocardiographers who had no information about the clinical details of each subject undertook transthoracic echocardiographic examinations using the Vivid E95® cardiac ultrasonography system (GE VingMed Ultrasound AS; Horten, Norway) with 2.5- to 5-MHz probes. EAT thickness was measured at the level of the right ventricular free wall in the parasternal long-axis view. Measurements were obtained at end-diastole, as this phase allows for maximal visualization of the epicardial fat layer. The EAT thickness was defined as the distance between the visceral layer of the pericardium and the outer wall of the myocardium. To improve accuracy and minimize variability, three consecutive cardiac cycles were averaged for each measurement. Interobserver and intraobserver variability for EAT measurements were assessed using intraclass correlation coefficients (ICCs) and Bland-Altman analysis, with ICC values of 0.91 and 0.94, respectively, indicating excellent reproducibility. The mean absolute difference between repeated measurements was 0.5 ± 0.2 mm for intraobserver variability and 0.6 ± 0.3 mm for interobserver variability.

The study protocol was approved by the Local Ethics Committee of Cumhuriyet University, and all procedures were conducted in accordance with the Declaration of Helsinki (2010-01/13). Written informed consent was obtained from all participants before enrollment. The study adhered to guidelines for the ethical conduct of research involving human subjects, and patient confidentiality was maintained throughout the study.

Statistical analysis

Statistical analyses were conducted using IBM SPSS Statistics for Windows, Version 25 (Released 2017; IBM Corp., Armonk, New York, United States). Continuous variables were presented as mean ± standard deviation (SD), while categorical variables were expressed as frequencies and percentages. The normality of the continuous data was assessed using the Kolmogorov-Smirnov test. For comparisons between two groups (patients with and without AF), the independent samples t-test was applied for normally distributed continuous variables, and the Mann-Whitney U test was used for non-normally distributed variables. Categorical variables were compared using the chi-square test or Fisher’s exact test, as appropriate. Receiver operating characteristic (ROC) curve analysis was performed to evaluate the predictive value of epicardial adipose tissue (EAT) thickness for AF. The area under the curve (AUC) was calculated to quantify the accuracy of EAT as a diagnostic marker, with an AUC value closer to 1 indicating excellent discriminative ability. Sensitivity, specificity, and the optimal cut-off value for EAT thickness were determined using the Youden Index, which maximizes the sum of sensitivity and specificity. A p-value of less than 0.05 was considered statistically significant for all analyses. Confidence intervals (CIs) were reported at the 95% level to indicate the precision of the estimates. Interobserver and intraobserver variability for EAT thickness measurements were assessed using ICCs, with ICC values above 0.80 indicating excellent reliability. This statistical approach was selected to ensure a robust analysis of the relationship between EAT thickness and AF while accounting for potential differences in baseline characteristics between study groups.

## Results

A total of 110 patients with HFpEF were included in the study, among whom 20 (18%) had AF and 90 (81%) did not. The baseline characteristics of the study population are summarized in Table 1. Patients with AF were comparable in age to those without AF (mean age: 66 ± 11 years vs. 69 ± 10 years, p = 0.160). The prevalence of hypertension was higher in the AF group compared to the non-AF group (70% vs. 63%, p = 0.129), and patients with AF were more likely to have diabetes mellitus (65.0% vs. 39%, p = 0.038). There was no significant difference in sex distribution between the two groups. EAT thickness was significantly greater in patients with AF compared to those without AF (8.3 ± 0.9 mm vs. 7.1 ± 0.8 mm, p < 0.001), as shown in Table 1. These findings suggest that increased EAT thickness is associated with the presence of AF in HFpEF patients.

**Table 1:** Baseline characteristics of study patients ALT: alanine aminotransferase; AST: aspartate aminotransferase; HDL: high density lipoprotein; LDL: low density lipoprotein; WBC: white blood count; AF: atrial fibrillation

	AF (+), n = 20	AF (-), n = 90	p-value
Age (years)	66 ± 11	69 ± 10	0.160
Gender (M/F)	12/8	58/32	0.378
Hypertension (%)	14 (70%)	57 (63%)	0.129
Diabetes mellitus (%)	13 (65%)	35 (39%)	0.038
Smoking (%)	5 (25%)	26 (29%)	0.776
Hyperlipidemia (%)	11 (55%)	41 (46%)	0.138
Left ventricular ejection fraction (%)	57 ± 6	59 ± 3	0.076
Left atrial diameter (cm)	4.6 ± 0.3	4.2 ± 0.5	< 0.001
Epicardial adipose tissue	8.3 ± 0.9	7.1 ± 0.8	< 0.001
Glucose (mg/dL)	115 (64-450)	122 (73-342)	0.333
Creatinine (mg/dL)	1.2 ± 0.6	1.3 ± 0.5	0.076
ALT, IU/L	20 (5-218)	28 (6-112)	0.328
AST, IU/L	22 (2-118)	29 (11-115)	0.057
Triglycerides (mg/dL)	131 (46-693)	118 (36-205)	0.061
Total cholesterol (mg/dL)	160 (96-287)	148 (49-276)	0.072
HDL cholesterol (mg/dL)	42 (23-66)	36 (12-62)	0.053
LDL cholesterol (mg/dL)	115 (58-221)	104 (39-215)	0.105
Hemoglobin (g/dL)	14 ± 2	12 ± 3	0.065
Platelet (10³/µL)	215 ± 63	224 ± 51	0.745
WBC (10³/µL)	8.1 ± 2	8.2 ± 2	0.955

**Figure 1:**
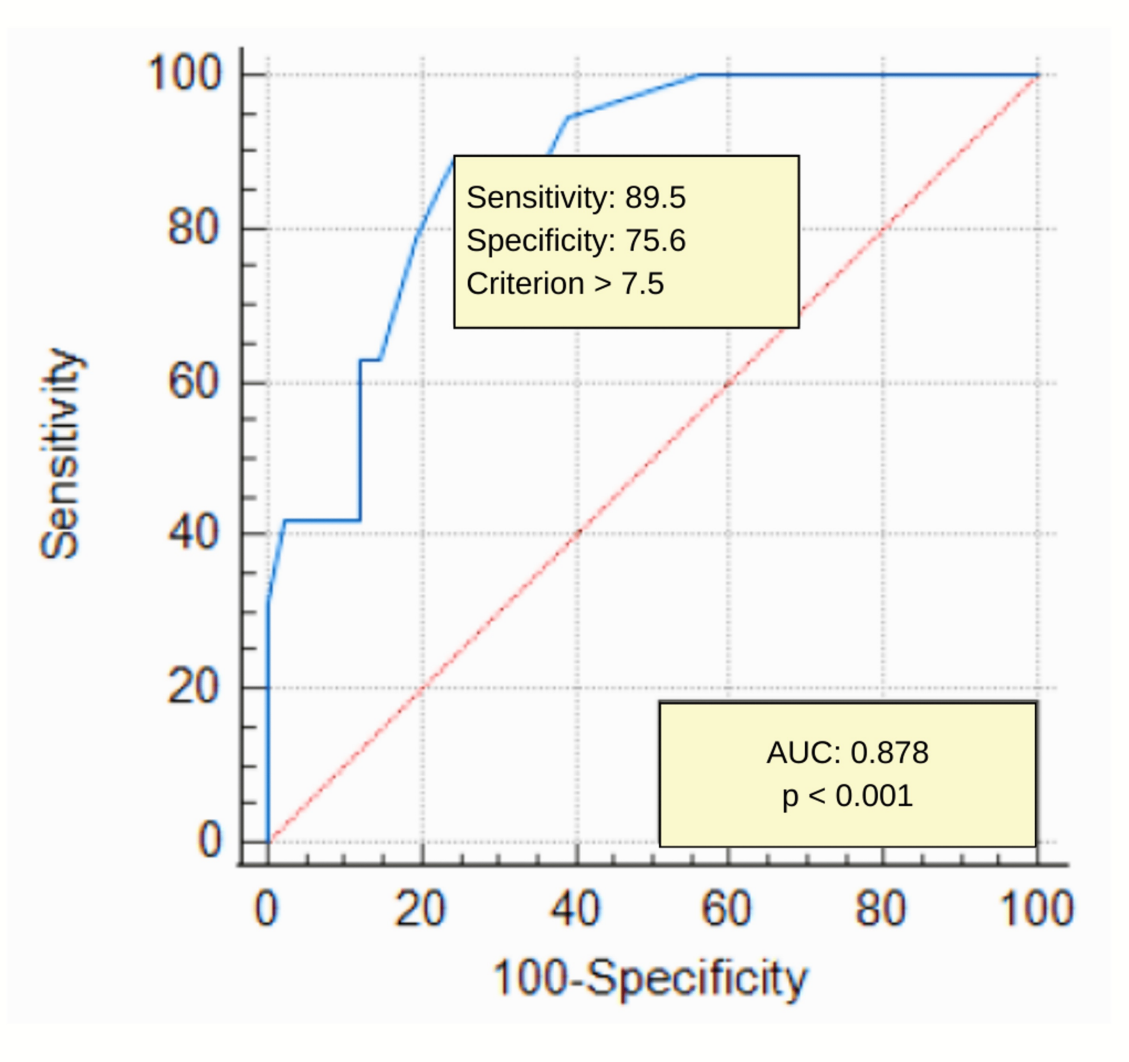
Receiver operating characteristic (ROC) analysis demonstrated that EAT thickness was a strong predictor of AF (AUC = 0.878, p < 0.001), with a cut-off value of 7.5 mm achieving 89% sensitivity and 75% specificity EAT: epicardial adipose tissue; AF: atrial fibrillation; AUC: area under the curve

ROC analysis was performed to evaluate the ability of EAT thickness to predict the presence of AF. The analysis revealed an AUC of 0.878 (95% CI: 0.76-0.93, p < 0.001), indicating excellent diagnostic performance. An optimal cut-off value of 7.5 mm for EAT thickness was identified, which provided a sensitivity of 89% and a specificity of 75% (Figure 1). These results highlight the potential of EAT thickness as a robust, non-invasive biomarker for AF in HFpEF patients. To ensure the reliability of EAT thickness measurements, interobserver and intraobserver variability were assessed using ICCs. The ICC for interobserver variability was 0.87, and the ICC for intraobserver variability was 0.89, demonstrating excellent reproducibility of the measurements. These findings suggest that EAT thickness is strongly associated with AF in HFpEF and may serve as a practical tool for identifying patients at higher risk of AF in clinical practice.

## Discussion

This study demonstrates a significant association between EAT thickness and the presence of AF in patients with HFpEF. EAT thickness was significantly higher in patients with AF, and ROC analysis confirmed its strong predictive value for AF, with an optimal cut-off value of 7.5 mm achieving excellent sensitivity and specificity. These findings contribute to the growing body of evidence linking EAT, a metabolically active fat depot, to cardiovascular pathophysiology, particularly in the context of AF and HFpEF.

Epicardial adipose tissue and atrial fibrillation

EAT is a visceral fat depot located between the myocardium and the visceral pericardium. Unlike subcutaneous fat, EAT has direct contact with the myocardium, sharing the same microcirculation and lacking a separating fascia. This unique anatomical relationship allows EAT to exert paracrine and vasocrine effects on the myocardium and adjacent cardiac structures [18]. Pro-inflammatory cytokines secreted by EAT, such as interleukin-6, tumor necrosis factor-α, and monocyte chemoattractant protein-1, are implicated in promoting atrial fibrosis, structural remodeling, and electrical instability, all of which are key mechanisms underlying AF [19,20]. The findings of this study align with previous research demonstrating a link between increased EAT thickness and the prevalence or severity of AF. Leggio et al. showed that EAT thickness was significantly higher in patients with AF compared to those in sinus rhythm, with a strong correlation between EAT volume and AF burden [21]. Mahajan et al. provided further evidence by demonstrating that EAT contributes to atrial structural remodeling, including increased atrial fibrosis and conduction slowing, which are critical substrates for AF maintenance [22]. In the context of HFpEF, where systemic inflammation and left atrial remodeling are already prominent, the additive effect of EAT may exacerbate the propensity for AF.

Epicardial adipose tissue in heart failure with preserved ejection fraction: a unique pathophysiological context

HFpEF is characterized by systemic inflammation, endothelial dysfunction, and myocardial stiffening, all of which contribute to diastolic dysfunction and elevated left atrial pressures [23,24]. These processes are further amplified by comorbidities such as obesity, diabetes, and hypertension, which are prevalent in HFpEF and contribute to the expansion of EAT [25]. Obesity, in particular, is closely linked to increased EAT deposition, as demonstrated in studies showing that EAT volume correlates strongly with BMI and waist circumference [26,27]. In HFpEF patients with AF, the role of EAT may be even more pronounced. Elevated left atrial pressures and stretching, common in HFpEF, create a substrate for atrial remodeling. The pro-inflammatory cytokines and profibrotic mediators secreted by EAT may amplify these effects, promoting atrial fibrosis and electrical remodeling. Furthermore, the increased EAT thickness observed in this study supports the hypothesis that EAT may serve as both a marker and a mediator of atrial remodeling in HFpEF patients with AF.

Clinical implications

The findings of this study highlight the potential utility of EAT thickness as a non-invasive biomarker for identifying HFpEF patients at higher risk of developing AF. Transthoracic echocardiography, a widely available imaging modality, can be used to measure EAT thickness, making it a practical tool in routine clinical practice. The optimal cut-off value of 6.5 mm identified in this study provides a threshold that may guide clinicians in risk stratification and early intervention. From a therapeutic perspective, interventions targeting EAT may hold promise for reducing AF risk in HFpEF. Lifestyle modifications, such as weight loss and exercise, have been shown to reduce EAT volume and improve atrial remodeling, as evidenced by the LEGACY trial, which demonstrated significant reductions in AF burden with weight management [28]. Pharmacological approaches targeting inflammation, such as the use of sodium-glucose cotransporter-2 (SGLT2) inhibitors or glucagon-like peptide-1 (GLP-1) receptor agonists, may also reduce EAT volume and its adverse effects on the myocardium [29]. Further research is warranted to explore the impact of these interventions on clinical outcomes in HFpEF patients with AF.

Strengths and limitations

This study has several strengths, including its focus on HFpEF, a population with limited data on the role of EAT, and the use of transthoracic echocardiography, a non-invasive and widely accessible imaging method, for EAT thickness measurement. The strong statistical significance of the findings and the excellent reproducibility of EAT measurements further enhance the reliability of the results. However, the study also has limitations. The cross-sectional design precludes conclusions about causality between increased EAT thickness and AF. Additionally, the study was conducted at a single center with a relatively small sample size, which may limit the generalizability of the findings. The lack of a volumetric EAT assessment, which could provide more comprehensive insights into the relationship between EAT and AF, is another limitation. Despite these limitations, the study provides important insights into the pathophysiological role of EAT in HFpEF patients with AF and sets the stage for future prospective and interventional studies.

Future directions

Future research should focus on longitudinal studies to establish causal relationships between EAT and AF in HFpEF. Interventional trials exploring the impact of targeted therapies, such as anti-inflammatory agents or weight loss programs, on EAT thickness and clinical outcomes in HFpEF patients with AF, are also needed. Advanced imaging modalities, such as cardiac magnetic resonance imaging or computed tomography, could be used to assess EAT volume and distribution more precisely, providing additional insights into its pathophysiological role.

## Conclusions

In conclusion, this study demonstrates that increased EAT thickness is strongly associated with the presence of AF in patients with HFpEF. EAT thickness measured by transthoracic echocardiography is a practical, non-invasive biomarker that may aid in risk stratification and guide therapeutic interventions in this population. The findings underscore the need for further research to explore the role of EAT as a modifiable risk factor and a therapeutic target in HFpEF patients with AF.
